# Do COVID-19 pandemic-related behavior changes affect perioperative respiratory adverse events in children undergoing cardiac interventional catheterization?

**DOI:** 10.1186/s12871-022-01951-8

**Published:** 2022-12-28

**Authors:** Wei Ji, Kan Zhang, Mengqi Li, Siyuan Wang, Liping Sun, Yue Huang, Jie Bai, Mazhong Zhang, Jijian Zheng

**Affiliations:** 1 Department of Anesthesiology, Shanghai Children’s Medical Center & National Children’s Medical Center, affiliated to Shanghai Jiao Tong University School of Medicine, Shanghai, 200127 China; 2 Pediatric Clinical Pharmacology Laboratory, Shanghai Children’s Medical Center & National Children’s Medical Center, affiliated to Shanghai Jiao Tong University School of Medicine, Shanghai, 200127 China; 3 Department of Anesthesiology 3201 Hospital, Xi’an Jiaotong University Health Science Center, Hanzhong, 723000 Shaanxi Province China

**Keywords:** COVID-19, Peri-operative respiratory adverse events, Congenital heart disease, Children, Upper respiratory infection

## Abstract

**Background:**

The novel coronavirus disease (COVID-19) suddenly broke out in China in December 2019. Pandemic-related behavioral changes can cause perioperative respiratory adverse events in children with congenital heart disease (CHD). Here, we compared the incidence of perioperative respiratory adverse events (PRAEs) in CHD children with and without upper respiratory infection (URI) undergoing the cardiac catheterization before and during the COVID-19 pandemic.

**Methods:**

This prospective observational single-center study was based at a tertiary care center in Shanghai, China. A total of 359 children with CHD with and without recent URI were included between January 2019 and March 2021. The overall incidence of PRAEs (laryngospasm, bronchospasm, coughing, airway secretion, airway obstruction, and oxygen desaturation) in non-URI and URI children undergoing elective cardiac catheterization was compared before and during the COVID-19 pandemic. A logistic regression model was fitted to identify the potential risk factors associated with PRAEs.

**Results:**

Of the 564 children enrolled, 359 completed the study and were finally analyzed. The incidence of URIs decreased substantially during the COVID-19 pandemic (14% *vs.* 41%, *P* < 0.001). Meanwhile, the overall PRAEs also significantly declined regardless of whether the child had a recent URI (22.3% *vs.* 42.3%, *P* = 0.001 for non-URI and 29.2% *vs.* 58.7%, *P* = 0.012 for URI, respectively). Post-operative agitation in children without URI occurred less frequently during the pandemic than before (2.3% *vs.* 16.2%, *P* = 0.001). Behaviors before the COVID-19 pandemic (odds ratio = 2.84, 95% confidence interval [CI] 1.76–4.58) and recent URI (odds ratio = 1.79, 95% CI 1.09–2.92) were associated with PRAEs.

**Conclusions:**

COVID-19 pandemic-related behavioral changes were associated with a reduction in PRAEs in non-URI and URI children undergoing elective therapeutic cardiac catheterization.

## Key points

- The incidence of URIs in children undergoing elective cardiac interventional catheterization decreased substantially during the COVID-19 pandemic period.
- The overall PRAEs incidence, Especially the lower SpO2, also significantly decreased in the non-URI and URI children with CHD.
- The COVID-19 pandemic-related behavioral changes can reduce perioperative respiratory adverse events in children undergoing cardiac interventional catheterization, Both with and without URI.

## Introduction

More than 18 months have passed since the first case of the novel coronavirus disease (COVID-19) was reported on December 1, 2019, and this disease has not yet been fully controlled [1, 2]. COVID-19 transmission can result in severe respiratory disease, which can lead to hospitalization and death; however, shifts in routine behavior in the public, such as social distancing, less time outdoors, mask wearing, and increased attention to hygiene were done to reduce transmission [3–8]. Although the disease has been brought under control after 76 days in China, behavioral changes remain [9]. It has been reported that the disease spectrum at the Respiratory Department and Infection Department in pediatric hospitals in China has dramatically changed due to the reduction of pediatric upper respiratory infections (URI), which may be attributed to COVID-19 pandemic-related behavioral changes [4, 10]. The effects of the COVID-19 pandemic-related behavioral changes on clinical practice and patient outcomes remain unknown.

Determining the optimal timing to deliver anesthesia to children with URI can be challenging for both anesthesiologists and parents [11, 12]. The presence of URIs in children undergoing general anesthesia significantly increases perioperative respiratory adverse events (PRAEs) and may also increase the length of hospital stay, cost, suffering, and even death [13–16]. Postponement of surgery due to sudden URI may disturb both the surgeon’s, guardian’s, and children’s schedules, potentially even missing the optional surgical time window since children usually experience URI 7–8 times each year, and it is better to delay the surgery for 4–6 weeks in children with URI scheduled for general anesthesia to reduce PRAEs [17].

This is particularly true for children with congenital heart disease (CHD), since they are more vulnerable to URI than otherwise healthy children [18, 19]. Furthermore, the incidence and severity of post-operative respiratory complications significantly increased in children with CHD undergoing open-heart surgery [20]. The length of stay (LOS) in the hospital and cardiac intensive care unit (ICU) is also significantly 2–3 times longer in children with URIs than non-URI [20]. Therefore, open heart surgery in children with URIs should be postponed whenever possible.

Compared to open heart surgery, therapeutic cardiac catheterization is relatively less invasive and has a lower incidence of life-threatening events [21, 22]. However, our previous study showed that the incidence of overall PRAEs in children with less than 2 weeks of URI undergoing cardiac catheterization remains very high, approximately 66.3% compared to 46.6% in non-URI children [12]. Along with the ongoing COVID-19 pandemic-related behavioral changes, whether these behavioral changes can affect the incidence of PAREs in non-URI and URI children undergoing cardiac interventional catheterization remains unknown. Thus, the present study aimed to investigate the incidence of PRAEs in non-URI-affected children during the COVID-19 pandemic. The secondary endpoint was to examine whether the overall PRAEs were also decreased in children with URIs, and whether the URI and/or COVID-19 pandemic were potential risk factors for PRAEs.

## Materials and methods

### Ethics

This prospective study (SCMCIRB-K20170122) was approved by the Institutional Review Board of Shanghai Children’s Medical Center, China (Chairperson Prof. Fan Jiang) on June 30, 2017, and was registered with the Chinese Clinical Trial Registry (ChiCTR2000034531).

### Pediatric patients

The included data were extracted from our database, as previously described. Children scheduled for elective cardiac transcatheter occlusion under general anesthesia for ventricular septal defects, atrial septal defects, and/or patent ductus arteriosus were recruited into the study from February 20, 2019, to March 19, 2021, at the Shanghai Children’s Medical Center and National Children’s Medical Center, a tertiary hospital affiliated with Shanghai Jiao Tong University. Exclusion criteria included parents’ refusal to sign the informed consent; the American Society of Anesthesiologists physical status score ≥ IV; evidence of a recent lower respiratory tract infection (such as pneumonia and bronchitis) within the past 2 weeks; unavailable medical history (parents or legal guardian cannot recall the medical history clearly); known hypersensitivity to anesthetics; recent participation in other clinical studies; medical history of hepatic or nephritic disease or complex cyanotic heart disease; premedication (e.g., dexmedetomidine, salbutamol, and midazolam); and endotracheal tube in exchange for LMA due to unsatisfactory ventilation.

### Anesthesia management

After the children entered the operating room, routine monitoring including electrocardiography, noninvasive blood pressure monitoring, and pulse oxygen saturation measurements were conducted. Anesthetics such as propofol (3–4 mg/kg) and sufentanil (0.1–0.2 μg/kg) were used for the induction of anesthesia and were administered through the peripheral vein. An appropriate LMA was inserted when the child was unconscious. Sevoflurane 1–1.2 MAC with a mixture of oxygen and air (50:50) was inhaled to maintain the depth of anesthesia, depending on the heart rate and blood pressure. Crystalloid fluid was infused according to 4–2-1 rule. The LMAs were removed while the children were still anesthetized (regular breathing, end-tidal sevoflurane level: 0.8 MAC). The anesthesiologist in charge was blinded to the patient’s history of URIs.

### Protocol

All children with CHD were divided into two groups: the COVID-19 period from March 2020 to March 2021 and the non-pandemic period from February 2019 to January 2020, with January 23 being the date when Wuhan declared strict anti-pandemic measures that would cause changes in behavior.

Before surgery, the parents or legal guardians of all children were visited by a senior resident anesthesiologist with at least three years of anesthesia experience, and the parents/guardians were asked to fill out a questionnaire form. The questionnaire included questions concerning the patients’ demographic information (age, sex, weight, and height), type of CHD, history of asthma and passive smoking, presence of URI symptoms, and the exact time of URI occurrence. Patients who presented with any two of the following URI symptoms, as confirmed by the parent or legal guardian, over the last two weeks were considered to have a history of URI: nasal congestion, rhinorrhea, dry or moist cough, sore throat, sneezing, or fever > 38 °C.

PRAEs (laryngospasm, bronchospasm, coughing, airway secretion, airway obstruction, and oxygen desaturation) and details of anesthesia management were recorded. Adverse respiratory events were defined as any episode of perioperative airway obstruction (snoring or requirement of intervention with a decrease in SpO_2_ after inhalation of room air; interventions including repositioning/neck roll, jaw thrust/chin lift, and oral airway), laryngospasm (partial or complete airway obstruction associated with muscle rigidity of the abdominal and chest walls), bronchospasm (increased respiratory effort, especially during expiration; wheezing on auscultation), oxygen desaturation less than 95% (for ≥ 10 s), breath-holding (≥ 15 s), severe coughing (a series of pronounced, persistent, severe coughs lasting more than 10 s), and increased airway/oral secretion (≥ one suction). Intraoperative observation and postoperative visits were completed by a qualified anesthesiologist in our study team. If laryngospasm occurred, the children were treated with positive airway pressure combined with increased anesthetic levels; some patients required the administration of succinylcholine. In cases where bronchospasm occurred, the children were treated with nebulized albuterol using a metered-dose inhaler.

### Statistical analysis

According to our previous studies [12, 23], the incidence of PRAEs in children with CHD without recent URI was 37.7% (95% confidence interval [CI] 35.3% to 40.2%) during the non-COVID-19 pandemic period. We assumed that the incidence of PRAEs in the same population would decrease by half in 2020 (the COVID-19 period). A total of 114 children were required in each period with a power of 90% and a significance level of 0.05. Accounting for a 10% dropout of samples, 125 patients per group were required.

Normality testing was conducted using the Shapiro–Wilk test. Numerous variables are presented as means and standard deviations (SD), and categorical data are presented as absolute numbers and percentages. Differences between groups were determined using Student’s t-test for numerous variables and the χ^2^ test or Fisher’s exact test for categorical variables. A logistic regression model was used to measure the association between behavioral changes during the pandemic and PRAEs. The results were presented as odds ratios (OR) and 95% CI. For all tests, a two-sided value of *P* value < 0.05 was considered significant. Statistical analyses were performed using IBM SPSS Statistics for Windows, version 26.0 (IBM Corp., Armonk, NY, USA).

## Results

**Fig. 1:**
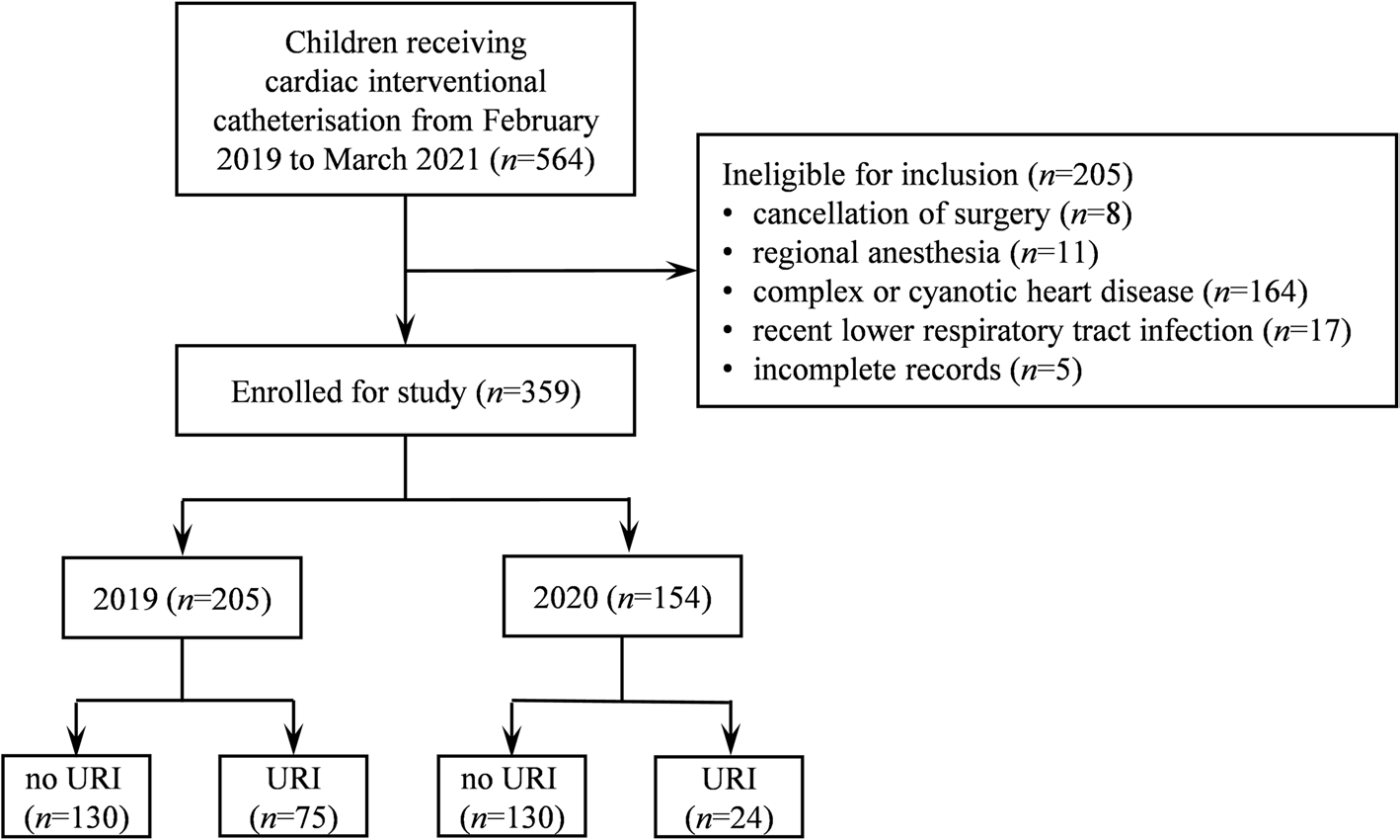
Flow diagram

Of the 564 pediatric patients recruited, 364 were eligible for inclusion in the observational study. Finally, 359 children with complete records were analyzed: 260 before and 154 during the COVID-19 pandemic. A STROBE flow diagram is shown in Fig. 1. During the COVID-19 pandemic, only 15.6% of children were diagnosed with recent URI, which was significantly lower than before (vs. 36.6%, *P* < 0.001). Demographic characteristics were comparable before and during the COVID-19 period (Table 1), except for passive smoking, which occurred less frequently during the pandemic.

**Table 1:** Characteristics of all children. Data were shown as mean ± SD or *n* (%). ^†^ was considered as statistically significant (*P* < 0.05)

	non-URI		*P* value	URI		*P* value
	2019 *n* = 130	2020 *n* = 130		2019* n* = 75	2020 *n* = 24	
age, year	3.1 (2.2, 5.2)	3.3 (2.1, 6.4)	0.219	2.7 (2.1, 4.4)	3.6 (2.3, 6.0)	0.076
gender, *n*			0.900			0.919
M	56 (43)	57 (44)		29 (38.7)	9 (37.5)	
F	74 (57)	73 (56)		46 (61.3)	15 (62.5)	
height, cm	97 (88, 110)	100 (87, 121)	0.174	95 (88, 106)	100 (90, 125)	0.172
weight, kg	15 (12, 19)	16 (12, 23)	0.078	14 (12, 18)	15 (13, 22)	0.127
tobacco exposure, *n*	44 (33.8)	12 (9.2)	< 0.001^†^	30 (40.0)	2 (8.3)	0.004^†^
history of asthma, *n*	2 (1.5)	1 (0.8)	1.000	1 (1.3)	0 (0)	NA
history of allergy, *n*	12 (9.2)	12 (9.2)	1.000	19 (25.3)	3 (12.5)	0.188
history of hay fever, *n*	10 (7.7)	6 (4.6)	0.302	8 (10.7)	4 (16.7)	0.477
snoring, *n*	38 (29.2)	13 (10.0)	< 0.001^†^	35 (46.7)	9 (37.5)	0.432

**Fig. 2:**
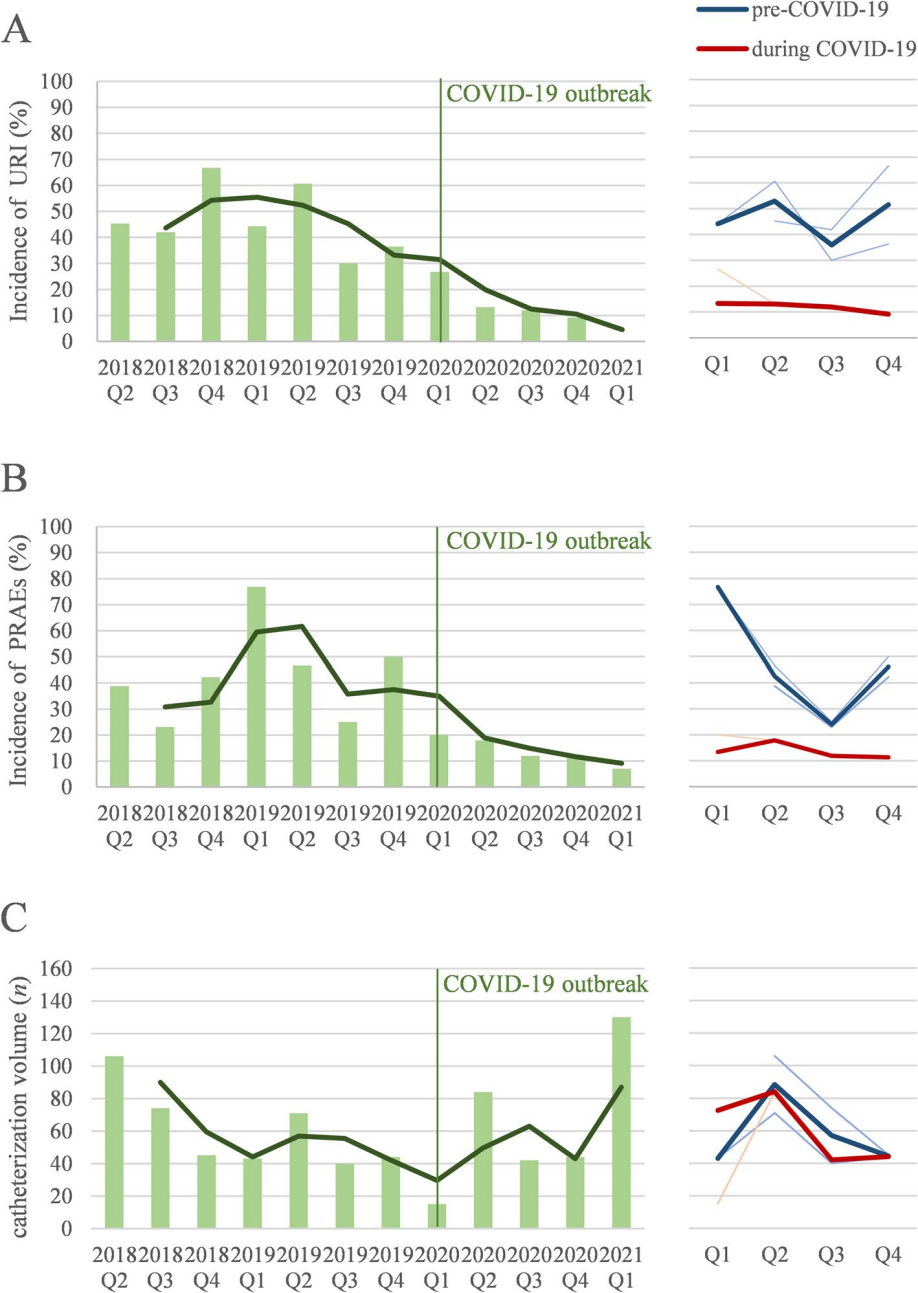
The left panels show a quarterly trend for the incidence of upper respiratory infection (URI, panel **A**) and perioperative respiratory adverse events (PRAEs, panel **B**), and the total surgery volume (panel **C**) from the 2nd quarter (Q2) of 2018 to 1st quarter of 2021. The green line is the moving average showing the chronological order of the incidence and volume. The right panels show the seasonality of URI, PRAEs, and surgical volume. The blue line is the average line before the pandemic (2018 and 2019), whereas the red line represents the line during the pandemic

The overall volume of cardiac catheterization was similar in 2019 and 2020, with a dramatic decrease in quarter 1 (Q1) 2020 when the COVID-19 outbreak and the Chinese health authorities declared traffic restriction (Fig. 2 and Table 2). The overall incidence of URI and PRAEs before the pandemic showed a seasonal trend. Along with the decrease in URI in the consecutive time from Q1 2019 to Q1 2021, the overall incidence of PRAEs also demonstrated a similar downward trend. During the COVID-19 pandemic, PRAEs in all children decreased by 50% compared with the same period in 2019. In children without URI, the overall PRAEs decreased from 42.3% to 22.3% (*P* = 0.001). In children with URIs, it decreased from 58.7% to 29.2% (*P* = 0.012). The difference in perioperative lower SpO_2_ levels was particularly clear before and during the pandemic. Post-operative agitation occurred less frequently in non-URI children during the pandemic than before (2.3% *vs*. 16.2%, *P* = 0.001).

**Table 2:** Incidence of peri-operative respiratory adverse events and post-operative adverse events. Data were shown as *n* (%). ^†^ was considered as statistically significant (*P* < 0.05). NA, not applicable

	non-URI		*P* value	URI		*P* value
	2019 *n* = 130	2020 *n* = 130		2019* n* = 75	2020 *n* = 24	
PRAEs, *n*						
severe cough	2 (1.5)	1 (0.8)	1.000	0 (0)	0 (0)	NA
breath holding	0 (0)	1 (0.8)	NA	0 (0)	0 (0)	NA
laryngospasm	5 (3.8)	0 (0)	NA	3 (4.0)	1 (2.4)	1.000
airway obstruction	28 (21.5)	16 (12.3)	0.047^†^	34 (45.3)	18 (25.0)	0.077
desaturation	48 (36.9)	20 (15.4)	< 0.001^†^	37 (49.3)	4 (16.7)	0.005^†^
any of above	55 (42.3)	29 (22.3)	0.001^†^	44 (58.7)	7 (29.2)	0.012^†^
Others, *n*						
agitation	21 (16.2)	3 (2.3)	0.001^†^	6 (8.0)	0 (0)	NA
vomiting	9 (6.9)	7 (5.4)	0.606	4 (5.3)	1 (4.2)	1.000
copious secretion	8 (6.2)	5 (3.8)	0.393	11 (14.7)	3 (12.5)	1.000
fever	2 (1.5)	1 (0.8)	1.000	2 (2.7)	1 (4.2)	0.569

Univariate analysis indicated that three variables were associated with PRAEs (Table 3). Multivariate analysis identified pandemic-related behavioral changes as an independent factor that mitigated the risk of PRAEs (OR 0.33, 95% CI 0.21 to 0.52; *P* < 0.001). Recent URI were associated with an increased incidence of PRAEs (OR 1.79, 95% CI 1.09 to 2.92, *P* = 0.02).

**Table 3:** Multivariate analysis of factors associated with perioperative respiratory adverse events (PRAEs). Results from logistic regression were presented as mean ± SD, *n* (%), odds ratio (OR) with 95% confidence interval (95% CI) and Wald test *P* value. ^†^ was considered as statistically significant (*P* < 0.05)

	univariate analysis			multivariate analysis	
	PRAEs (*n* = 135)	non-PRAE (*n* = 224)	*P* value	OR (95% CI)	*P* value
age, year	3.5 ± 2.5	4.2 ± 2.8	0.089		
gender, *n*			0.404		
M	53 (39)	98 (44)			
F	82 (61)	126 (56)			
year, *n*			< 0.001^†^		
2019	99 (73)	106 (47)		2.84 (1.76, 4.58)	< 0.001^†^
2020	36 (27)	118 (53)		Reference	
URI, *n*			0.002^†^		
non-URI	84 (62)	176 (79)		Reference	
URI	51 (38)	48 (21)		1.79 (1.09, 2.92)	0.020^†^
tobacco exposure, *n*	43 (32)	45 (20)	0.012^†^		
history of snoring, *n*	43 (32)	52 (23)	0.072		
history of hay fever, *n*	11 (8)	17 (8)	0.848		
history of asthma, *n*	3 (2)	1 (0.4)	0.151		

## Discussion

We found that the incidence of URIs in children undergoing elective cardiac interventional catheterization decreased substantially during the COVID-19 pandemic period. The overall PRAEs incidence also significantly decreased in non-URI and URI children with CHD, especially in those with lower SpO_2_. This decrease in PRAEs may be seen as a benefit from the behavioral changes during the COVID-19 pandemic period and provides a potential pathway to reduce the PRAEs incidence and medical costs and increase safety through behavioral changes before surgery.

Unexpectedly, our current study showed that PRAEs were significantly reduced in children with CHD with and without URI compared to our data before the COVID-19 pandemic outbreak. Along with many other studies, our previous studies also demonstrated that recent URI significantly increased PRAEs in children undergoing elective cardiac interventional catheterization and postpone of surgery was thought as an easy and effective way to reduce PRAEs [12, 14, 23]. Although the precise mechanisms remain elusive, multivariate regression analysis indicated that COVID-19 pandemic-related behavioral changes were associated with these reductions in PRAEs, and these behavioral changes might be adopted as an effective strategy for pre-operative preparation for reducing PRAEs in children with CHD regardless of URI. Wearing a mask as a non-pharmaceutical intervention is an effective measure to prevent the transmission chain of the virus [4]. Wearing facemasks offered several benefits, but did not completely block the occurrence of PRAEs. A combination of interventions will always be required to provide effective protection from airway infection, reduce higher airway sensitivity, and reduce PRAEs [10, 24].

The study showed a steep decline in PRAE in Q3 followed by a steep rise, which may be related to the decreasing surgery volume after the autumn semester began and the lower incidence of URI in Q3. Children with URI increased while the seasons switched from winter to spring (Q2) and from autumn to winter (Q4).

In contrast to our previous study that passive smoking is an independent risk factor for PRAEs in children with URI [23], our current study showed that the percentages of passive smoking in non-URI and URI children were dramatically reduced and passive smoking was no longer an independent risk factor after the COVID-19 pandemic breakout, which might be related to social distancing and/or smoking reduction in guardians due to COVID-19 pandemic-related behavior changes. Reduction of tobacco exposure might alleviate airway stimulation and finally reduce PRAEs in CHD children, which further investigation is needed to clarify it.

In addition to PRAEs reduction, post-operative agitation was alleviated after the COVID-19 pandemic. Due to the sharp decrease in operation volume during the COVID pandemic, anesthesiologists and residents were able to provide sufficient care for the emergence and post-operative recovery of each patient. Furthermore, under the “one ward for one child” policy in order to reduce in-hospital transmission, post-operative crying and noise from a crowded place with many people nearby disappeared. PRAEs reduction is also a potential cause of agitation alleviation.

Our study also indirectly confirmed a recent report from Taiwan showing a significant decrease in cases of influenza, enterovirus, and all-cause pneumonia during the COVID-19 period, which might be due to the influence of voluntary and policy-related behavioral changes, including improved personal hygiene, less environmental tobacco exposure, social distancing and school closing, and restrictions on transportation and movement [7]. Although our data demonstrated seasonal changes in URI and PRAEs in the consecutive years of 2018 and 2019 with the lowest incidence in Q3, the incidences of URI and PRAEs substantially decreased during the COVID-19 pandemic without significant seasonal changes. After the COVID-19 outbreak, there was no difference in the incidence of PRAEs between URI and non-URI children (29.2% vs. 22.3%, *P* = 0.466).

### Strengths and limitations

A key strength of our study is that the data were prospectively collected instead of retrospective analysis only because this was a part of our case series study [12, 23, 25], which increased the ability to trace the effects of COVID-19 pandemic-related sudden behavioral changes on PRAEs occurrence in children undergoing elective cardiac interventional catheterization. However, our study has some limitations. First, COVID-19 is a sudden outbreak of pandemic disease, making it impossible for us to conduct a cohort study or randomized controlled trial to explore its potential effects on PRAEs; thus, we only collected data prospectively and analyzed them retrospectively. Second, several voluntary and policy-induced behavioral changes have occurred due to the COVID-19 pandemic, but the specific or primary behavioral change related to PRAEs reduction was not identified in the current study, which is very important for future changes in pre-operative preparation strategies. Third, the mechanisms of COVID-19 pandemic-related behavioral changes induced by PRAEs reduction have not been explored. Fourth, the results drawn from this single-center database may not be generalizable to patients nationally or to other geographic contexts.

## Conclusions

The COVID-19 pandemic-related behavioral changes can reduce perioperative respiratory adverse events in children undergoing cardiac interventional catheterization, both with and without URI. Whether and how these behavioral changes could be adopted as an effective strategy for pre-operative preparation to reduce PRAEs occurrence is worthy of further investigation.

## Data Availability

The datasets used and/or analyzed during the current study are available from the corresponding author upon reasonable request.

## Abbreviations

COVID-19: Novel coronavirus disease
CHD: Congenital heart disease
PRAEs: Perioperative respiratory adverse events
URI: Upper respiratory infection
LOS: Length of stay
ICU: Intensive care unit
LMA: Laryngeal mask airway
MAC: Minimum alveolar concentration
CI: Confidence interval; OR, odds ratio
